# The Effect of Cataract on Early Stage Glaucoma Detection Using Spatial and Temporal Contrast Sensitivity Tests

**DOI:** 10.1371/journal.pone.0128681

**Published:** 2015-06-08

**Authors:** Johann Klein, Barbara K. Pierscionek, Jan Lauritzen, Karin Derntl, Andrzej Grzybowski, Margarita B. Zlatkova

**Affiliations:** 1 School of Biomedical Sciences, University of Ulster, Cromore Road, Coleraine, BT521SA, United Kingdom; 2 Faculty of Science, Engineering and Computing, Kingston University, Penrhyn Road,Kingston-upon-Thames, KT1 2EE, United Kingdom; 3 Ordination Dr. Karin Derntl, Ophthalmologist, Wartenburgerstr. 1b, 4840 Vöcklabruck, Austria; 4 Department of Ophthalmology, Poznań City Hospital, Poznań, Poland; 5 Department of Ophthalmology, University of Warmia and Mazury, Olsztyn, Poland; University of Montreal, CANADA

## Abstract

**Background:**

To investigate the effect of cataract on the ability of spatial and temporal contrast sensitivity tests used to detect early glaucoma.

**Methods:**

Twenty-seven glaucoma subjects with early cataract (mean age 60 ±10.2 years) which constituted the test group were recruited together with twenty-seven controls (cataract only) matched for age and cataract type from a primary eye care setting. Contrast sensitivity to flickering gratings at 20 Hz and stationary gratings with and without glare, were measured for 0.5, 1.5 and 3 cycles per degree (cpd) in central vision. Perimetry and structural measurements with the Heidelberg Retinal Tomograph (HRT) were also performed.

**Results:**

After considering the effect of cataract, contrast sensitivity to stationary gratings was reduced in the test group compared with controls with a statistically significant mean difference of 0.2 log units independent of spatial frequency. The flicker test showed a significant difference between test and control group at 1.5 and 3 cpd (p = 0.019 and p = 0.011 respectively). The percentage of glaucoma patients who could not see the temporal modulation was much higher compared with their cataract only counterparts. A significant correlation was found between the reduction of contrast sensitivity caused by glare and the Glaucoma Probability Score (GPS) as measured with the HRT (p<0.005).

**Conclusions:**

These findings indicate that both spatial and temporal contrast sensitivity tests are suitable for distinguishing between vision loss as a consequence of glaucoma and vision loss caused by cataract only. The correlation between glare factor and GPS suggests that there may be an increase in intraocular stray light in glaucoma.

## Introduction

The pernicious nature of glaucoma and sight loss that this disease can cause renders early diagnosis vital. Visual field losses caused by glaucoma can occur with no signs or symptoms. Yet pathogenesis of the disease is not fully understood [1]. The ability to detect contrast has relevance to the progression of glaucoma and is measured using contrast sensitivity tests that assess the extent to which this ability has been lost. Since contrast sensitivity testing for sinusoidal grating patterns was introduced as a research tool [2,3], studies have shown that grating contrast sensitivity has the potential to detect early glaucoma [4–8], is a better predictor of visual function than visual acuity [9,10], discriminates between glaucoma and/or other ocular disease and normal subjects [11–13] and thus has the potential to be an efficient screening tool for detecting glaucoma at its early stages [12,14,15]. There is also substantial evidence to indicate that ability to detect flickering targets which measures temporal contrast sensitivity may be significantly reduced before visual field defects can be detected or before changes in the optic nerve become apparent [7, 16–19].

Although standard automatic perimetry (SAP) which is used to assess visual fields remains the main psychophysical test used for measuring visual field loss in glaucoma, it does not consider the anatomical arrangement of nerve fibres [20,21,22] and has a high degree of non-stationary test-retest variability which is dependent on the sensitivity and on the type of analysis used (ie a Full Threshold strategy or the Swedish Interactive Threshold Algorithm (SITA)) [22].

Tests of temporal contrast sensitivity i.e. flicker perimetry have been used in a battery of tests for glaucoma [6,19,23–24] and inspired the development of the frequency doubling technology (FDT) perimeter [25]. Temporal contrast sensitivity is considered more resistant to the effects of lens opacities [26–28]. Yet, it has been suggested recently that the high temporal frequency stimuli in FDT render this more susceptible to reduced retinal illumination when light is attenuated in opacified or older lenses [29]. In this study the ability of spatial and temporal contrast sensitivity tests to detect early glaucoma in the presence of cataract was investigated using a control group matched for cataract type and severity. Contrast sensitivity for spatio-temporal stimuli, were investigated to find what combination of parameters may allow detection of glaucoma in the presence of cataract.

## Methods

### Subjects

**Fig 1:**
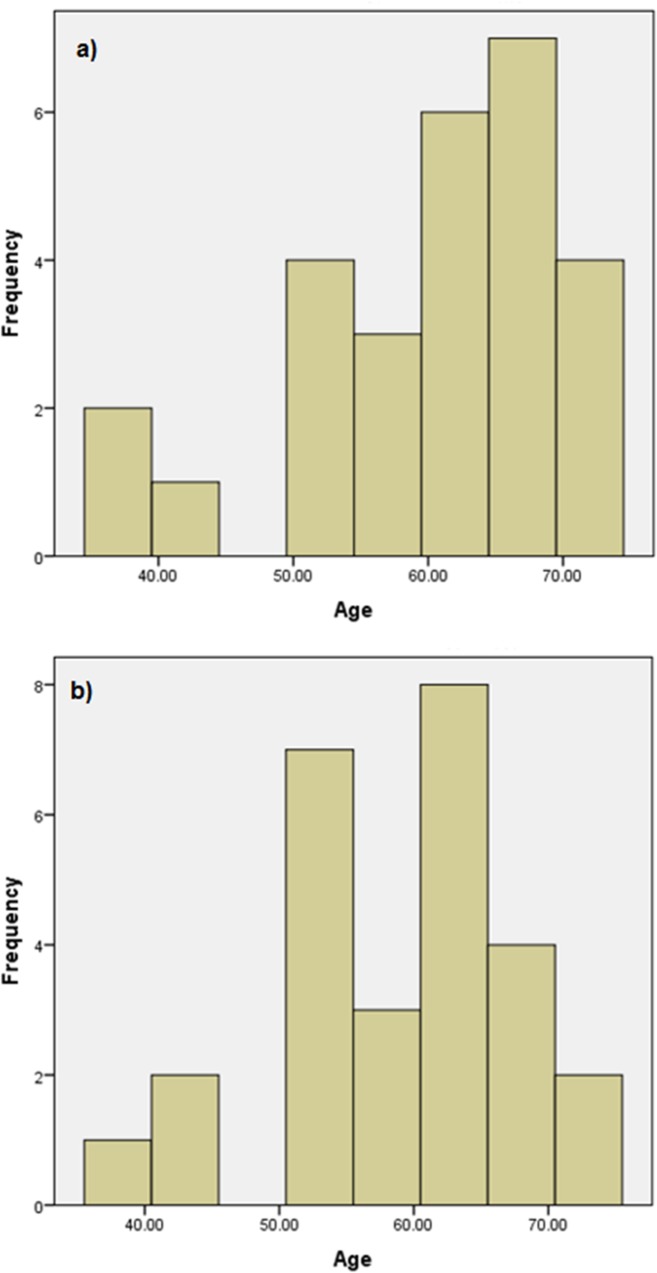
Frequency histogram of age distribution. The age distribution is shown for a) test subjects (with glaucoma and cataract: mean 60 years, SD 10.22 years) and b) controls (cataract only: mean 59 years, SD 9 years). Each cohort comprised 27 subjects.

Subjects were recruited from a clinical practice in Upper Austria. Twenty-seven subjects with early glaucoma who presented with low grade or no glaucomatous field losses with simultaneous early cataractous signs were selected together with twenty-seven control subjects matched for age and cataract type and severity (S1 and S2 Tables). The mean ages of the test and control groups were 60 ± 10.2 (SD) years (ranging from 37 to 74 years, 17 females, 10 males) and 59.03 ± 9.0 years (ranging from 38 to 73 years, 14 females, 13 males) respectively. The age distribution of the test and control cohorts is shown in Fig 1.

### Clinical examination

All subjects underwent an ophthalmological examination including a full medical history, refraction, dilated fundus examination (two drops tropicamide 0.5%) with a 90 dioptre lens and the Heidelberg Retinal Tomograph (HRT, Heidelberg Engineering, Germany), slit lamp biomicroscopy of the anterior segment of the eye and Goldmann contact tonometry. All subjects had a best corrected Snellen Visual Acuity of 20/30 or better. Refractive errors were between ±4 diopters spherical equivalent and less than 1.25 diopters cylinder. The pupil diameter was in the range of 4–5 mm. Corneal thickness was measured with an optical coherence pachymeter (OCP mobile, 4Optics, Germany). Subjects who suffered from diabetes, were frail or had any history of ocular or neurological disease other than glaucoma or cataract were excluded from the study. Data for all subjects is shown in S1 and S2 Tables).

**Fig 2:**
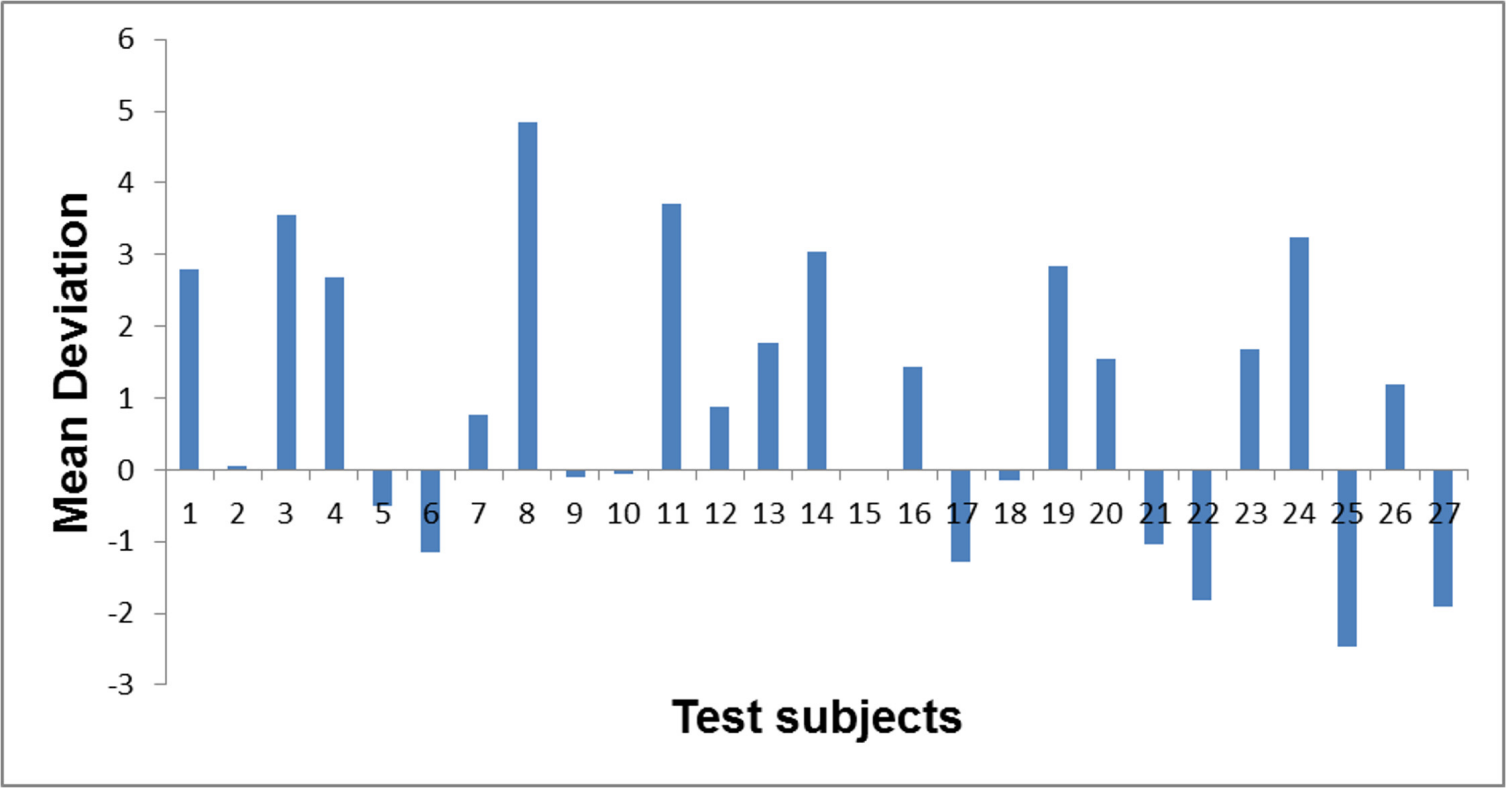
Mean deviation for test subjects. The mean deviation (MD), a measure of visual field function, taken using the Oculus Centerfield 2 visual field analyzer using the glaucoma 30–2 fast threshold algorithm and the Goldmann size III target stimulus for test subjects ie those with glaucoma and early cataract. The stimulus colour was white, its duration was 200ms and the stimulus interval was 600ms. A negative value indicates that the MD is better than age-matched norms.

All subjects in the test group had a history of intraocular pressure (IOP) exceeding 21mmHg on two or more occasions as measured using Goldmann contact tonometry. The definitive diagnosis of glaucoma (by KD) was made on the basis of IOP values ≥21mmHg before treatment and abnormalities of the retinal fibre layer and optic nerve head: cup/disc ratio >3, thinning of the neuroretinal rim, changes in blood vessel direction and any evidence of disc hemorrhaging within a disc diameter of the optic nerve head. Visual field measurements were performed with Oculus Centerfield 2 visual field analyzer (Oculus, Inc, USA) using the glaucoma 30–2 fast threshold algorithm and the Goldmann size III target. The instrument uses a scale from 0-38dB. Reference luminance was 318cd/m^2^ and background luminance was 10cd/m^2^. The stimulus colour was white, the stimulus duration was 200ms and stimulus interval was 600ms. The average mean deviation (MD) of the subjects in the test group was –1.9 (ranging from –2.47 to 4.85), which is, among other criteria, indicative of early glaucoma according to the European Glaucoma Society Guidelines (EGS) [30]. Fig 2 shows the MDs for all test subjects; negative values indicate a value of MD that is better than age-matched norms. It should be noted that this instrument differs from the convention used in other perimeters for which positive values of MD are better than age-matched norms. Four of the test subjects had localized field defects within the central 10 degrees as determined by the total defect; one of these subjects also had a localized defect on the pattern deviation plot.

Structural parameters (rim area and volume, cup shape, height variation contour and mean retinal fibre layer thickness) were obtained from objective measurements of optic disc topography by HRT III. All test subjects received treatment either with latanoprost/timolol/dorzolamid or brinzolamid and at the time of testing IOP was controlled to < 21 mm.

Cataract grading was based on a scheme that grades both cortical and nuclear cataracts from 1 to 4 depending on cataract severity [31]. In the glaucoma and control groups, the grading ranged from 0.5 to 3 (nuclear) and 1 to 2 (cortical). Grading was conducted by two observers independently (KD and JK) and agreement found in all cases. The grading system is akin to the LOCS III [32]: nuclear cataract graded on colour and cortical opacification evaluated in accordance with opacity extension [31].

In the control group, 19 of the 27 subjects had nuclear and/or cortical opacities at the lowest grading level, 6 had either nuclear or cortical cataract at grade 2, one subject had both nuclear and cortical cataracts at grade 2 and one subject had a nuclear opacification at grade 3 and cortical opacity at grade 2. In the test group, 23 out of 27 subjects had nuclear and/or cortical opacities at the lowest grade, three subjects had grade 2 nuclear or cortical opacities, one subject had grade 2 nuclear and cortical cataract and one subject had grade 3 nuclear and grade 2 cortical opacities.

### Stimuli and apparatus

**Fig 3:**
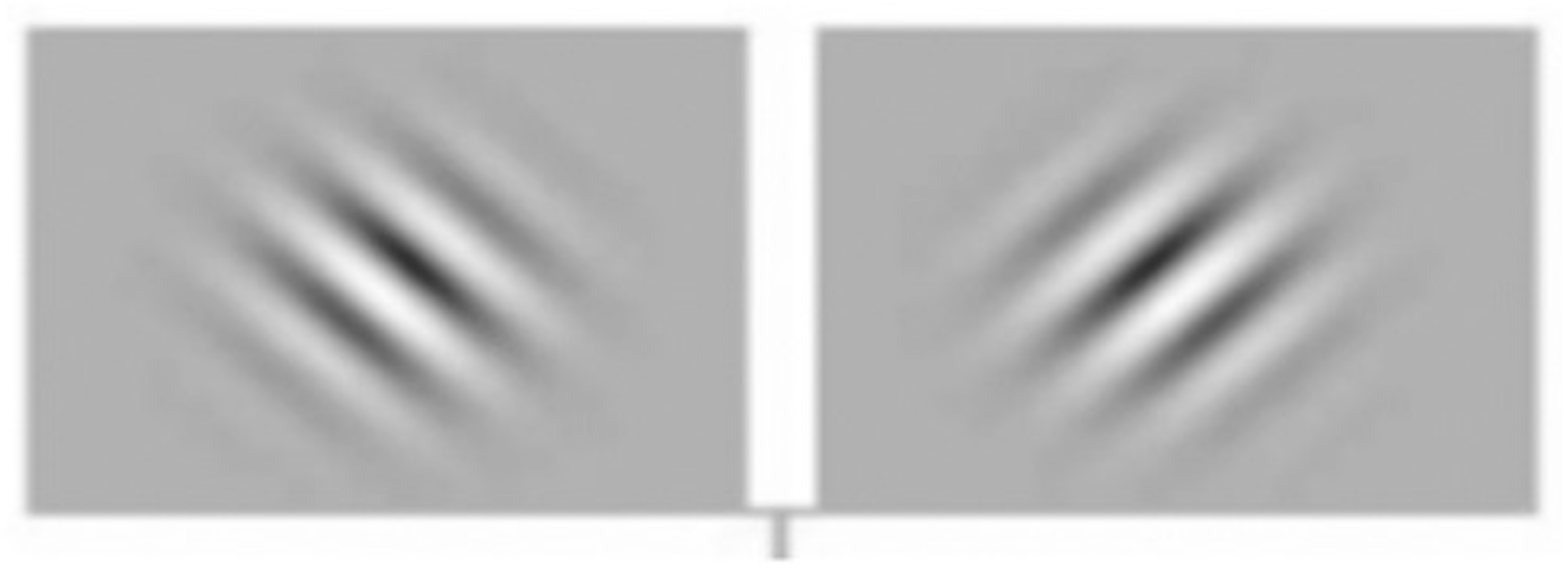
Example of the sinusoidal stimuli used in the experiments. The grating stimuli were circular Gabor patches of 2 degree standard deviation with sinusoidal luminance profile. The peak spatial frequencies tested were 0.5, 1.5 and 3 cycles/degree

Stimuli were circular gratings or Gabor patches with a sinusoidal luminance profile presented within a two dimensional Gaussian envelope of 2 deg standard deviation with around 95% of the contrast energy falling within a circular area which was 8 degrees in diameter. The Gabor stimuli had peak spatial frequencies of 0.5, 1.5 and 3cpd. An example of the stimuli used is shown in Fig 3.

Stimuli had the same mean luminance as the background (5.50 cd/m^2^) and were generated by a computer with an 8 bit graphic card and a TFT monitor (LG Flatron L227 WT). The monitor was gamma corrected and the luminance was measured, before each test, with a photometer (Spectrascan PR-650, Photo Research, Inc, CA, USA) to control output linearity. A glare source was used, consisting of four fluorescent tubes (Type OSRAM Dulux L 24W/840) placed around the four sides of the monitor [33]. Fluorescent, rather than bright, sources were proposed for testing glare in order to avoid afterimages and photostress effects [33]. The luminance of the glare source was 2700 cd/m^2^ which created a noticeable decrease in contrast sensitivity.

### Procedure

Contrast sensitivity for stationary gratings, was measured foveally with and without glare in both test and control groups. The display was viewed monocularly and the contralateral eye was occluded. For each subject (test and control) the dominant eye was tested. A headrest was used to stabilize the head of each subject and to maintain a viewing distance of 133cm. Trial lenses were used for optimal correction. The subjects adapted to the ambient light levels in the examination room for 5 minutes. Fixation was central and all measurements, including HRT and contrast sensitivity, were completed in one session which lasted 35–40 minutes. A training session preceded the main experiment for familiarization with the stimuli and procedure. The training session was used to ensure that subjects were familiar with the procedure, to explain that response to the stimulus should be prompt and to determine range of response times. It was noted that all cases responses were within 2–3 seconds which is longer than any critical duration thresholds [34–36] and therefore did not require recalculation for variation in contrast energy which can occur if response times are shorter than the critical duration of the stimulus [34–36]. Trials were initiated by the investigator; subjects in both cohorts responded verbally to the stimulus to minimize delays that may occur if the subject’s response necessitated a mechanical reaction such as pressing a button. As with the training session all responses were within 2–3 seconds. The three spatial frequencies (0.5, 1.5 and 3.0 cpd) were tested in the same order in all subjects. The contrast threshold for these stimuli was measured using a two-alternative forced choice (2AFC) psychophysical procedure combined with a 3 down 1 up staircase method. Stimuli were presented either at 45° or 135° orientations and subjects were asked to recognize and respond verbally to indicate whether the grating was tilted to the left or right. Three reversals of the staircase were used to calculate threshold. The dynamic range for this test was -0.7 to -2.9 log(contrast). These measurements were subsequently repeated with the glare source on. This was conducted after measurements with flickering gratings (described below). The outcomes were compared with tomographic scans and with visual field measurements.

Measurement of contrast sensitivity with flickering gratings was undertaken by having the subject view a circular Gabor patch oriented at 45° that was split into two by a vertical diameter. One half of the patch flickered on and off at 20 Hz; the other half did not flicker. The psychophysical methods used were 2AFC and staircase procedures described above. The dynamic range of the test was -0.4 to -2.6 log(contrast). The task required of the subjects was to determine which half of the stimulus was flickering and to indicate this verbally. The trials were initiated by the experimenter; subjects in both cohorts responded to the stimulus within 2–3 seconds. The contrast sensitivity was measured for the same spatial frequencies as above. No glare source was used. All other experimental conditions for testing contrast sensitivity with stationary and flickering gratings were identical.

### Statistical analysis

The summary statistics included calculations of mean values and 95% confidence intervals based on standard error of the mean. A 3-way ANOVA was performed to assess the statistical significance of the main effects of glaucoma, glare and stimulus spatial frequency on contrast sensitivity and to assess the statistical significance of their interactions. Pearson correlation was used to assess the association between contrast sensitivity and perimetric sensitivity or the optic nerve structural parameters. Non-parametric analysis was performed to compare distributions for test subjects and controls using chi-square test and contingency tables. The criterion for statistical significance was set at 0.05. All calculations were performed using SPSS 17.

The study was approved by the Biomedical Sciences Ethics Filter Committee (University of Ulster). Informed written consent was obtained from all subjects. The study was performed in accordance with the Tenets of the Helsinki Declaration.

## Results

### Contrast sensitivity for stationary gratings (0 Hz) with and without glare

**Fig 4:**
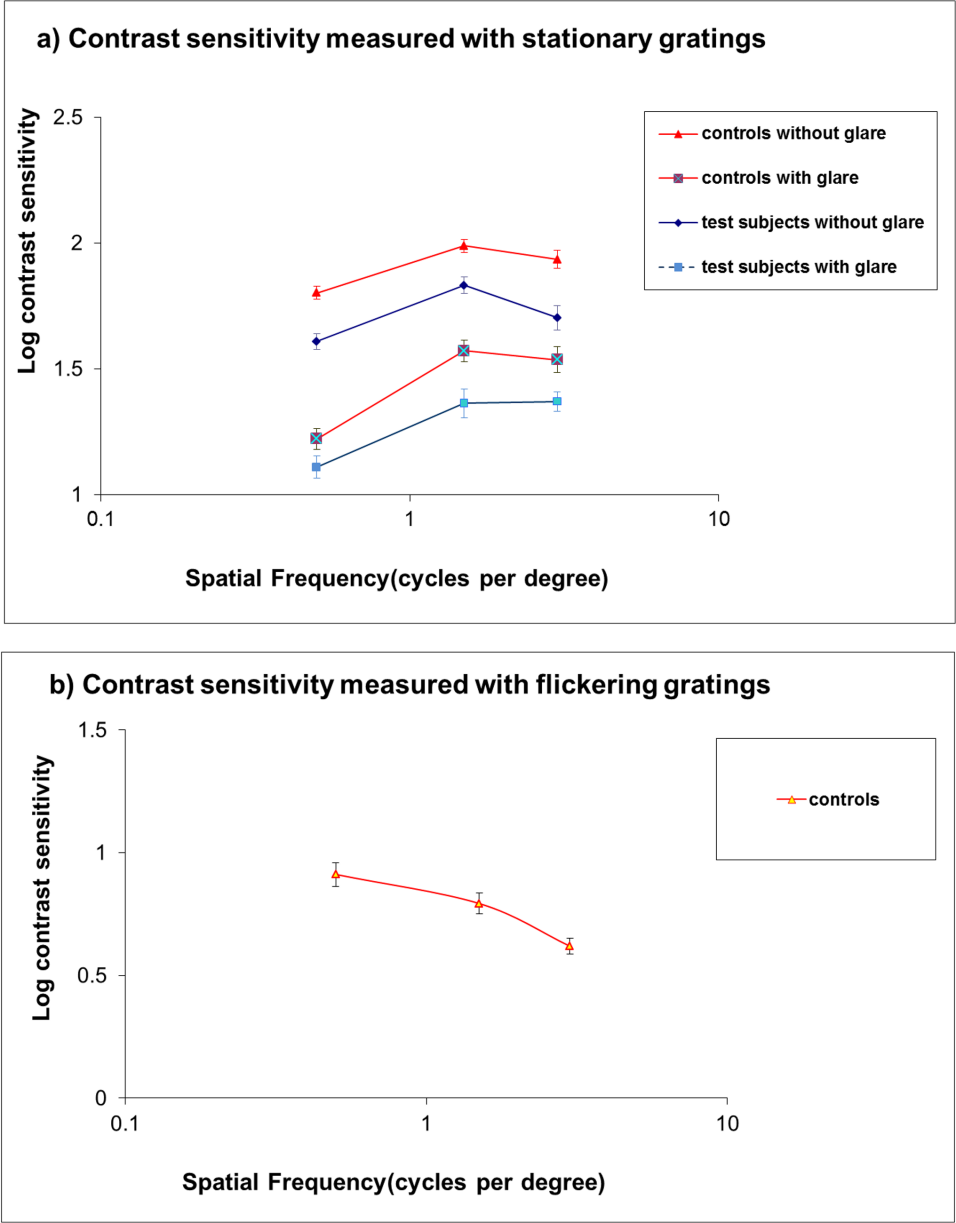
Contrast sensitivity for stationary and flickering gratings. a) Contrast sensitivity for stationary gratings (0Hz) for test subjects with glaucoma and early cataract compared with controls (cataract only); b) Contrast sensitivity for flickering gratings (20Hz) for controls with cataract only. Error bars represent 95% confidence intervals.

The average contrast sensitivity with and without glare for both groups is shown in Fig 4A). Overall reduction was found for test subjects relative to the controls. The presence of glare considerably reduced contrast sensitivity to a similar extent for both groups of subjects and was more pronounced at low spatial frequency, confirming previous findings [34]. The effects of glaucoma, glare and spatial frequency on contrast sensitivity were found to be statistically significant using 3-way ANOVA [F(1.305) = 53.9, p <0.0001; F(1.305) = 371.9, p<0.0001; F(2.305) = 46.5, p<0.0001, respectively]. The effect of glaucoma was similar at all spatial frequencies as indicated by the lack of interaction between the effects of glaucoma and spatial frequency [F(2.305) = 0.263, p = 0.769]. The two-way interaction between glare and spatial frequency was statistically significant [F(2.305) = 4.437, p = 0.013]. A statistically significant correlation (r = -0.456, 95% CI: -0.712 to -0.172) was found between the contrast sensitivity for test subjects at 0.5cpd and the MD. No statistically significant correlation was found between the MD and other spatial frequencies. There was a relationship between contrast sensitivity at 0.5cpd and rim area but this did not reach statistical significance (r = 0.197, p = 0.322). Correlation between the total rim area and visual field sensitivity in decibels (dB) and non-logarithmic units (1/lambert) were not statistically significant (r = 0.07, p >0.05 and r = 0.04, p>0.05 respectively). There was a slight increase in the correlation coefficient for central perimetric mean sensitivity and the temporal rim area but this was not statistically significant (r = 0.33, p>0.05 (dB); r = 0.21, p>0.05 (1/lambert), p>0.05).

**Fig 5:**
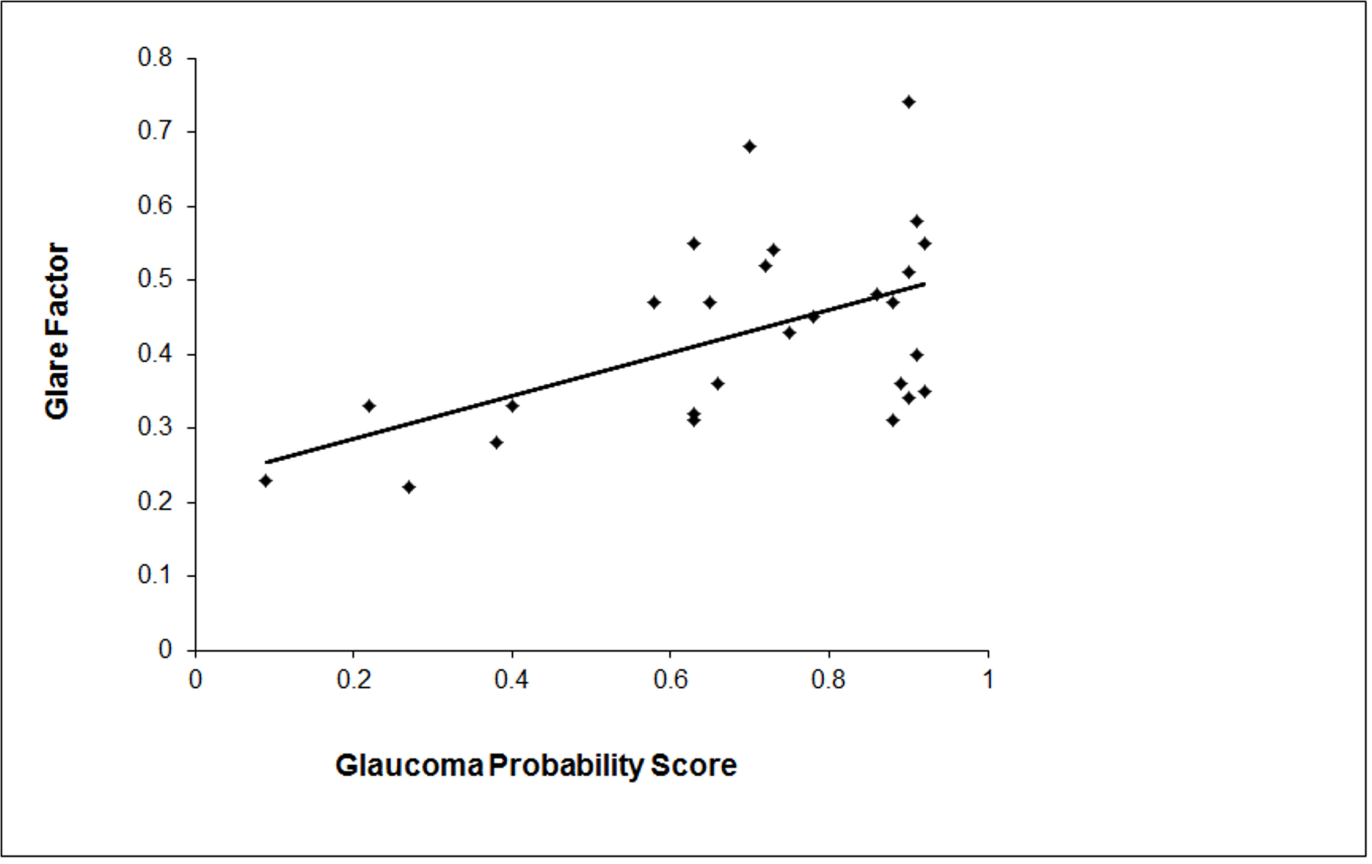
Glare factor plotted against Glaucoma Probability Score (GPS). The glare factor, which is the difference between log contrast sensitivity with glare and without glare, plotted against the Glaucoma Probability Score (GPS): an automated optic disc analysis system that shows the extent of damage to optic disc contour and topography caused by glaucoma. The correlation between glare factor and glaucomatous damage is statistically significant (ρ = 0.485 p = 0.01).

Fig 5 shows the relationship between the glare factor, calculated as the difference between log contrast sensitivity with glare and without glare, plotted against the Glaucoma Probability Score (GPS) (an automated system of optic disc analysis to ascertain the extent of glaucomatous damage based on disc contour and topography [37]) as assessed from the HRT III results. Spearman’s correlation showed statistical significance between glare factor and glaucomatous damage (ρ = 0.485 p = 0.01).

### Contrast sensitivity for flickering gratings: qualitative analysis

**Fig 6:**
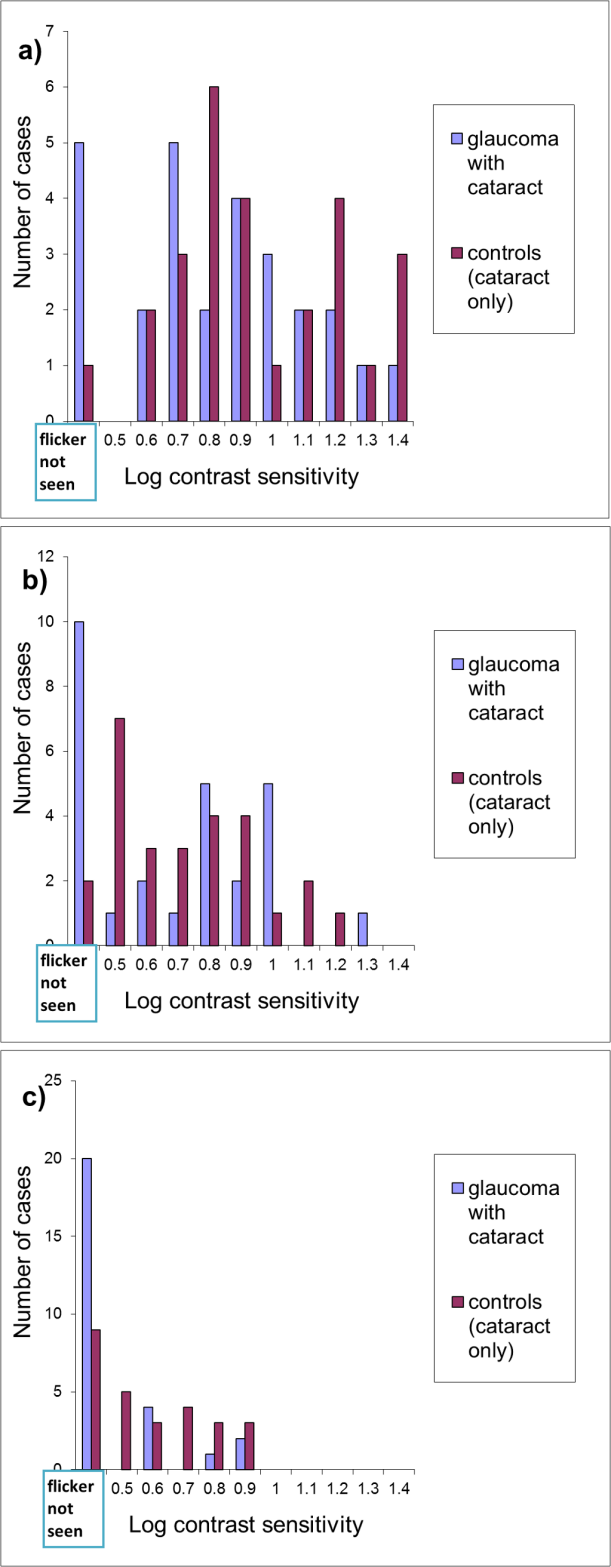
Distributions of flicker sensitivity. Histograms of the distributions of 20Hz flicker sensitivity at spatial frequencies of: a) 0.5 cpd; b) 1.5 cpd; and c) 3 cpd in test subjects with glaucoma and early cataract compared to controls with cataract only. Subjects who could not detect the flicker at all are represented by the leftmost column.

The results for controls are shown in Fig 4B). A number of test subjects could not detect the flickering grating even at the highest possible contrast ie -0.4log(contrast): at 1.5cpd and 3cpd, 10 and 20 test subjects, respectively, could not detect flicker. To allow comparisons with the flicker sensitivity in the control group, results are presented as flicker sensitivity distributions (Fig 6). Chi-square and contingency table analyses showed a statistically significant difference between the distributions for test subjects and controls at 1.5 and 3 cpd [χ^2^(2) = 9.75, p = 0.019 and χ^2^(2) = 9.17, p = 0.011 respectively] suggesting that glaucoma, in the presence of cataract, causes a substantial sensitivity loss for flicker compared with cataract only.

**Fig 7:**
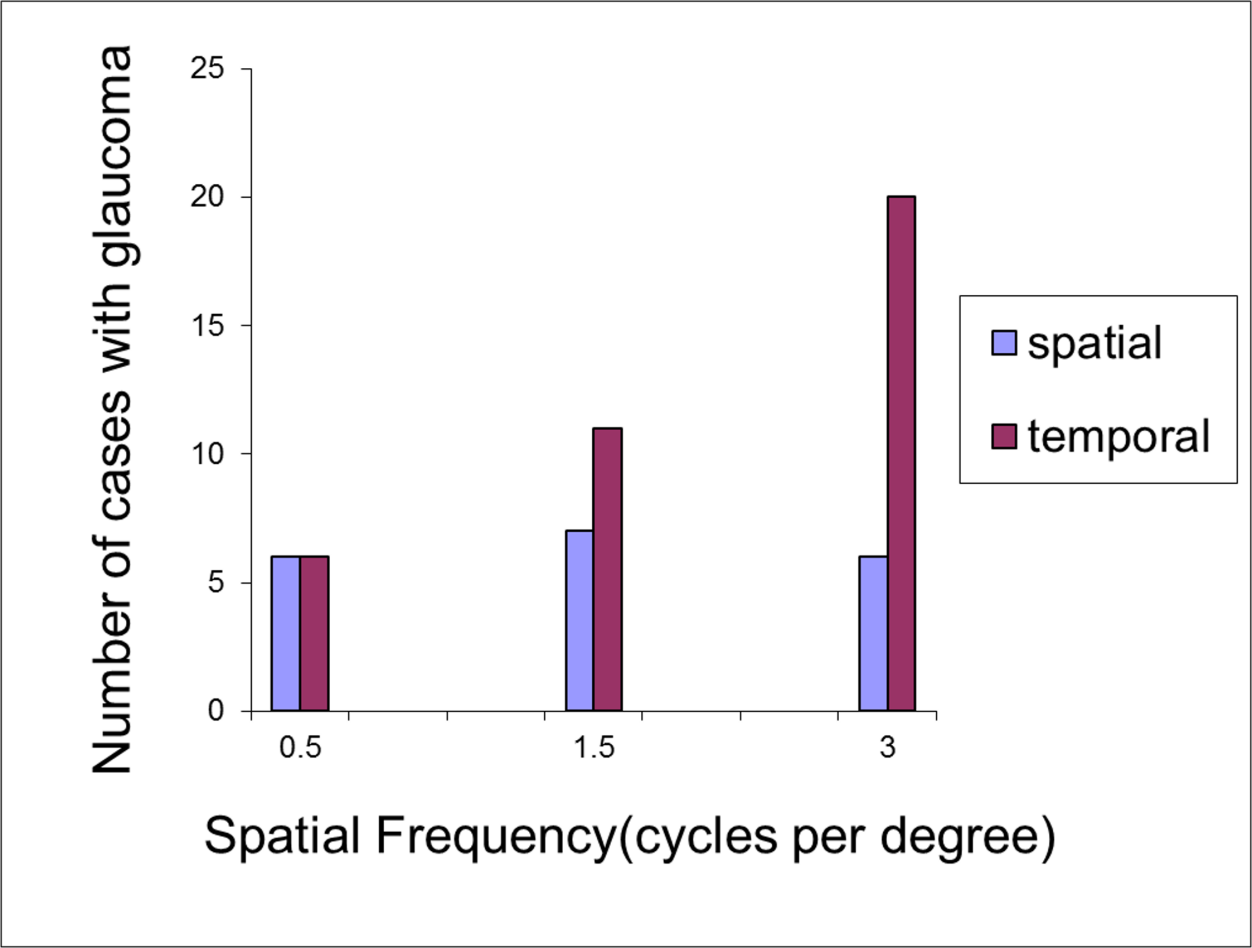
Number of test subjects with glaucoma and early cataract with significantly impaired contrast sensitivity. Bars represent the number of test subjects who fell below the 5^th^ percentile of contrast sensitivity responses of the control subjects (indicating statistically significant impairment), for stationary and flickering gratings.

Fig 7 compares the ability of the tests based on stationary and flickering gratings to differentiate between glaucoma with cataract from the presence of cataract alone. The number of test subjects who had contrast sensitivity values below the 5th percentile of the distribution of contrast sensitivity values for the control group was calculated for each test and plotted separately for each spatial frequency. At 3cpd, the number of test subjects who showed visual loss below the 5% limit of the controls was more than three times greater for the temporal contrast sensitivity test compared to the spatial test indicating the superior ability of the flicker test at 3cpd to differentiate between glaucoma with the presence of cataract compared with cataract alone.

## Discussion

This study aimed to determine if static and flicker contrast sensitivity tests are able to detect glaucoma in the presence of cataract. The results indicate that contrast sensitivity in both types of test is significantly reduced in subjects with glaucoma and cataract relative to subjects with cataract only. For stationary gratings, the loss was not dependent on spatial frequency. A lack of spatial frequency dependent selective loss in glaucoma has been described for gratings presented in the fovea both continuously [8,10] or transiently [7,10] under both photopic and mesopic conditions [10].

Temporal contrast sensitivity was measured using a grating that flickered at 20Hz, a rate which does not selectively affect sensitivity to any particular spatial frequency [38]. The greatest difference in temporal sensitivity between test and control subjects was found at 3cpd. The ability to detect temporal changes for this combination was severely impaired in subjects with glaucoma: 74% (20 out of 27 test subjects) had abnormal sensitivity (below the 5% limit of controls) for the flicker test at a spatial frequency of 3cpd compared to 22% of the same cohort showing impaired sensitivity for the static test at this spatial frequency. This observation concurs with reports in the literature about the existence of separate thresholds for flicker and pattern detection [39]. The flicker threshold has been found to be lower than the pattern threshold in subjects with glaucoma and ocular hypertension [17]. These differing thresholds have been considered as evidence for the operation of two separate neural mechanisms, associated with the magnocellular (M) and parvocellular (P) visual pathways. M-cells are larger than P-cells throughout the retina and, based on histological evidence of a selective reduction in number of large retinal ganglion cells in glaucoma, it has been suggested that M-cells are more susceptible to damage in early glaucoma [40,41]. However, subsequent physiological studies of the higher visual pathways did not confirm such selective losses of the magnocellular system at the level of the lateral geniculate nucleus [42,43] and no convincing psychophysical evidence has been found for such a hypothesis [7,10, 44–45]. The data from this study, suggesting early glaucomatous damage of the flicker-sensitive mechanism, cannot be easily reconciled to the notion of selective magnocellular damage: it is not clear why the most significant loss was observed for flickering gratings at 3 cpd but not at lower spatial frequencies which are also optimal for the M-pathway. Functional overlap of the two pathways could offer some explanation of why psychophysical studies could not detect selective losses of the magnocellular system [46,47] and it has been hypothesised that visual loss in glaucoma may be the consequence of not only a loss of certain sub-populations of optic nerve fibres but also because of a reduction in the sampling characteristics or redundancies of the various nerve fibre sub-populations [48]. Hence, glaucomatous change could be better detected from a reduction in redundancy of a sub-population of cells that have minimal redundancy than from a larger population of cells with higher redundancy but that may have incurred more cell losses [48]. It should also be remembered that M-cells constitute around 10% of the retinal ganglion cell population and therefore any losses that are borne would be more obvious than from a larger cell population.

The test subjects who had low contrast sensitivity for stationary gratings also has a greater visual field loss as characterized by the MD but this trend was significant only at 0.5 cpd. The correlation may result from the fact that both measures are affected by stray light. Previous studies have reported that spatial contrast sensitivity reduction and visual field loss in glaucoma are poorly correlated [10] even at corresponding areas of the visual field [49], suggesting that these two measures are mediated by different mechanisms. However, there is some evidence that SAP with a conventional size III stimulus can preferably stimulate M-cells [50]. Assuming that M-cells mediate the detection of low spatial frequency gratings, this finding might explain the correlation between 0.5 cpd and MD found in the present work. More importantly, contrast sensitivity loss for 0.5 cpd showed a trend with thinning of the neural rim. Although this correlation did not reach statistical significance, it suggests that contrast sensitivity could be a sensitive indicator of early glaucomatous loss in the presence of cataract. No statistical significance was found with visual field sensitivity and rim area.

Visual field test points from the Humphrey 24–2 test pattern have been related to the optic nerve head regions using retinal nerve fibre maps [51]. It has recently been shown that damage can occur in the macular region even in early stages of glaucoma [52]. This manifests as thinning of the retinal ganglion cell and inner plexiform layer and is greatest in the inferior retina [52]. Hood et al [52] have defined a macular vulnerability zone that describes an area of localised retinal nerve fibre layer thinning associated with macula damage. Other studies have also noted macular damage in the early stages of glaucoma [53–55] prompting the recommendation that 10–2 visual field tests be conducted routinely [52]. In general these findings might explain the reduction in central contrast sensitivity found in the present study and in previous work.

Perimetry was not conducted on control subjects. A number of studies have considered the effect of cataract on sensitivity to SAP [56–58] or FDT perimetry [59–64]. Using FDT perimetry, cataract was found to adversely affect MD [59,60] and reduce mean sensitivity [61] but not to alter pattern standard deviation (PSD) [59, 61]. Neither of the cohorts had subjects with very advanced cataracts; it has been shown that even with cataracts advanced enough to warrant extraction, the effect of cataract on visual fields measured with SAP is negligible [56]. Any distortions of size may have been expected with scatter of light around the axis, as may manifest with a posterior sub-capsular opacity [57]. This was not seen in any subject.

Simulating straylight with diffuse filters had an effect on FDT perimetry [59–61]; Bergin et al [64] showed that this applied when simulation was above 50% increase in straylight [64]. The effect of cataract-induced light scatter on contrast sensitivity measurements has been described previously [64–66]. Similar effects of glare were seen in both cohorts which was consistent with the fact that test and control groups were matched for cataract type and severity. The results also showed that glare measurements can be influenced by glaucoma: a significant association was found between the glare factor and the GPS (Fig 5). Sensitivity and specificity of the GPS were found to be low with respect to damage in small and large optic discs respectively [67]. Corneal thickness and refractive error, both of which could serve as measures of ocular size, showed no correlation with GPS in this study; the range of these parameters within the subject cohort was not sufficiently wide to incorporate eyes with very small and very large optic discs.

Glare from excessive light scatter reduces retinal image quality and manifests as a decrease in contrast sensitivity [68]. A possible explanation for increased glare in subjects with glaucoma could be the effect of cell shrinkage that has been found in experimental glaucoma [69]. Shrinkage will alter the regularity of cell junctions and, akin to what is seen in the lens when aggregates form, may lead to subtle refractive index fluctuations along the inner ocular surface. It is not implausible that such fluctuations could scatter light within the eye ball leading to a veiling glare.

Given the size of the stimuli used in this study (required for presentation of a sufficient number of cycles at the spatial frequencies used) the method cannot replace conventional perimetry for detection of small subtle abnormalities in the visual field. It can, however, lead to development of adjuncts to perimetry that can differentiate between the effects of cataract on early stage glaucoma in contrast sensitivity tests.

This study tested a wider range of spatial frequencies than that used in FDT extending to higher spatial frequencies at which the frequency doubling illusion is not observed. Stationary gratings were also investigated and the results suggest that the spatio-temporal combination of high frequency flicker (20 Hz) with 3cpd produces an optimal result for differentiating glaucoma from cataract. High frequency flicker may not be the singular factor that reduces contrast sensitivity in the presence of cataract. Further work is needed to understand why losses in flicker sensitivity were seen at higher but not at lower spatial frequencies as well as to probe any correlation between glare level and glaucoma.

## Supporting Information

S1 Table Data for all control subjects who participated in this study showing clinical findings and psychophysical results. (XLS)

S2 Table Data for all test subjects who participated in this study showing clinical findings and psychophysical results. (XLS)
